# A novel toothbrush with a thin-head, slender-neck and super-tapered bristles enhancing accessibility in hard-to-reach areas: a crossover randomized trial

**DOI:** 10.1186/s12903-024-04975-3

**Published:** 2024-10-05

**Authors:** Hyo-Jung Kim, Joo-Yeon Lee, Eun-Song Lee, Da-Mi Kim, Ui-Won Jung, Jae-Kook Cha, Baek-Il Kim

**Affiliations:** 1 Department of Preventive Dentistry & Public Oral Health, BK21 FOUR Project, Yonsei University College of Dentistry, 120-752, 50 Yonsei-ro, Seodaemun-Gu, Seoul, Republic of Korea; 2 Department of Periodontology, Research Institute for Periodontal Regeneration, Yonsei University College of Dentistry, 120-752, 50 Yonsei-ro, Seodaemun-Gu, Seoul, Republic of Korea

**Keywords:** Dental care, Dental implant, Molars, Oral hygiene, Toothbrushing

## Abstract

**Background:**

Toothbrushing is the most commonly used method to physically remove dental plaque. However, there are many areas of the mouth that are difficult to reach with a toothbrush. The type of toothbrush is a critical factor influencing the effectiveness of oral care. The purpose of the study was to evaluate a toothbrush with a thin head, slender-neck and super-tapered bristles to target hard-to-reach areas in the oral cavity for reducing dental plaque and gingivitis.

**Methods:**

This crossover study included 58 adults aged 20 years and older. All participants were randomly assigned to use test and control toothbrushes (the latter had a normal head and round bristles) for two 4-week phases. Participants brushed their teeth twice daily in their habitual manner. At the start and end of each phase the Silness-Lӧe plaque index (PI), Lӧe -Silness gingival index (GI) and bleeding-on-probing index (BOP) were assessed and performed plaque fluorescence tests using quantitative light-induced fluorescence technology.

**Results:**

After using the test toothbrush, PI, GI and BOP decreased by 25%, 30% and 48%, respectively (*P* < 0.05). For the rearmost molars, PI, GI and BOP decreased by 18%, 26% and 47%, respectively (*P* < 0.05). For the implants, GI and BOP decreased by 31% and 57%, respectively (*P* < 0.05). The plaque fluorescence tests indicated that after using the test toothbrush, the dental plaque area for the anterior teeth and the simple plaque score for the rearmost molars decreased by 25% (*P* < 0.05) and 14% (*P =* 0.527), respectively.

**Conclusions:**

The test toothbrush was markedly better than the control toothbrush at reducing dental plaque and gingivitis. In particular, the test toothbrush produced an excellent reduction in dental plaque and gingivitis for the rearmost molars and the implants.

**Trial registration:**

KCT0009669, retrospectively registered 02/08/2024.

**Supplementary Information:**

The online version contains supplementary material available at 10.1186/s12903-024-04975-3.

## Introduction

Brushing teeth is the most commonly used method to physically remove dental plaque, and proper brushing is fundamental to oral hygiene [1]. However, there are many areas of the mouth that are difficult to reach with a toothbrush due to the presence of various structures such as the buccal mucosa, tongue and prostheses. For example, the rearmost molars and the area around dental implants are not easily accessible to a toothbrush, making dental plaque control difficult in those areas. The previous study examining the distribution of dental plaque and gingivitis in the dental arch have consistently shown that plaque and gingival indexes are higher for the rearmost molars than for the anterior teeth, with the greatest dental plaque deposition and highest gingivitis scores being reported for the molars [2]. Patients also often have difficulty cleaning under and around narrow-necked implants with bulbous crowns [3].

The formation of biofilms around implants plays a crucial role in the onset and progression of peri-implant diseases and contributes to inflammation around implants [4]. Peri-implant disease is characterized by an inflammatory response in the tissues surrounding an implant and comes in two forms: peri-implant mucositis and peri-implantitis [4]. If left untreated, peri-implant mucositis can progress to peri-implantitis, which is the leading cause of implant failure due to bone loss [5]. Previous studies evaluating peri-implant disease found peri-implant mucositis in 48% of implants aged 9–14 years and in 59.6% of implants aged approximately 10 years [6, 7]. Such peri-implant mucositis is reversible with early intervention and dental plaque control [8, 9]. Using a toothbrush for patient-administered mechanical dental plaque control is recommended as the standard method for managing peri-implant mucositis [10]. However, conventional toothbrush designs may have limitations in effectively managing dental plaque around implants and the rearmost molars, necessitating the development of specialized toothbrushes for those areas.

The type of toothbrush is a critical factor influencing the effectiveness of oral care, along with brushing habits [3]. Toothbrushes have various sizes, shapes, textures and designs to accommodate variations in the oral anatomy between individuals [11]. Advancements in toothbrush design, including in bristle length and position, head size and shape, and handle length, have been reported to enhance oral health [12]. Research studies have explored the effectiveness of different toothbrush designs in reducing dental plaque and gingivitis [13–16]. A 30-day randomized clinical trial of tapered-bristle toothbrushes in patients with gingivitis found lower plaque and gingivitis scores compared with using end-rounded toothbrushes [13]. One in vitro study found that toothbrushes with tapered soft and flexible bristles, called “super-tapered” bristles, improved access to the interproximal and gingival margins compared with toothbrushes with end-rounded bristles, and were more effective at removing artificial plaque from the gingival margin [14]. Design features other than the bristle characteristics also influence the efficacy in dental plaque removal. Axe A et al. [15] reported that toothbrush features that affect dental plaque removal include the head size and the diameter, length and softness of the bristles. An in vitro robot model study found that using a toothbrush with a flexible-neck design reduced the forces exerted in the oral cavity and hence also the damage to the teeth and gum [16]. That study also found that toothbrushes with flexible necks were more effective than toothbrushes with rigid necks at removing dental plaque, especially on the interproximal surfaces.

Dental plaque is a complex oral microbial ecosystem, and imbalance of its constituent microorganisms can result in pathogenic dental plaque that induces various oral diseases. Preventing and managing oral diseases requires more than simply detecting the presence of dental plaque, instead focusing on detecting pathogenic dental plaque and predicting its pathogenicity [17]. Red fluorescence is a type of biofluorescence induced by light irradiation in the visible region that is caused by metabolites (porphyrins) secreted by oral bacteria, and it can be detected using quantitative light-induced fluorescence (QLF) technology to assess the presence of pathogenic dental plaque. It has been reported that red biofluorescence produced by dental plaque is closely related to the development of dental caries and gingivitis, and it can be used to assess the pathogenicity of dental plaque [18–20]. Despite these advantages, QLF technology has not yet been widely applied in evaluations of the clinical efficacy of toothbrushes in dental plaque removal. Therefore, in this study we aimed to quantitatively determine the efficacy of a newly developed toothbrush in reducing dental plaque and gingivitis by utilizing an oral camera based on QLF technology that measures biofluorescence.

The primary purpose of the present study was to evaluate the newly developed toothbrush with a thin head, a slender neck and super-tapered bristles, which facilitated access to hard-to-reach areas of the oral cavity to reduce dental plaque and gingivitis. In particular, the study focused on reducing dental plaque and gingivitis at the rearmost molar and the implant sites, which are difficult to reach with a conventional toothbrush. The secondary objective was to characterize the convenience of and satisfaction with using the newly developed toothbrush relative to using a conventional toothbrush. The null hypothesis was that there is no difference in the effect of both regular and newly developed toothbrushes on dental plaque and gingivitis.

## Materials and methods

### Subjects

This study was approved by the Institutional Review Board of Yonsei University College of Dentistry Hospital in terms of the ethical protection of human subjects (IRB No. 2-2020-0108). Healthy subjects aged 20 years and older who voluntarily expressed their willingness to participate were recruited. All participants received written and oral explanations of the purpose and methods of the study as well as its confidentiality and the ability to withdraw from it with consequences, and then signed an informed-consent form. The study followed CONSORT guidelines for clinical trials. This study was conducted at Yonsei University Dental Hospital between June and September 2022 and was in compliance with the Declaration of Helsinki.

The inclusion criteria were the presence of at least 20 natural teeth, at least 1 distal non-restored tooth, at least one implant, and gingivitis. The exclusion criteria were as follows: receiving scaling or root planing within the previous 4 weeks, presence of infectious diseases, having taken antibiotics within the previous month, using an antimicrobial mouthwash during the study period, being pregnant or breastfeeding, severe pathological findings for the oral tissues (e.g. oral cancer or signs of intraoral inflammation), severe periodontal disease or multiple caries, or at least five teeth in the mouth requiring immediate caries treatment.

### Sample size calculation

The sample size was calculated based on the differences in plaque index between the conventional and new toothbrushes using data from a previous study [21]. A total of 52 participants were required, with an additional allowance for an estimated dropout rate, leading to a target of 58 participants. The calculations were performed using G*Power 3.1, assuming an effect size of 0.54 derived from previous research, a significance level of 0.05, and a power of 95%.

### Study procedure

This study was designed as a randomized, single-blind, crossover clinical trial. The study was conducted in two phases separated by a 4-week washout period. Participants were initially randomized at baseline (phase 1) to one of the two types of toothbrushes: a regular toothbrush (control group) or a newly developed toothbrush (test group). For 4 weeks each participant brushed their teeth in their habitual manner at least twice a day using the assigned toothbrush and the provided toothpaste. During this period, they were not allowed to use any other oral care products such as dental floss, interdental brushes or mouthwash. Participants were also instructed not to visit a dentist for any treatments (cavity treatment, periodontal treatment or preventive care) and were not allowed to take antibiotics.

The 4-week phase 1 trial was followed by a 4-week washout period. During the washout period, all participants were asked to use their usual toothbrush and toothpaste without any instructions. In phase 2 the control group was reassigned to using the newly developed toothbrush and the test group was reassigned to using the regular toothbrush. All participants then brushed for a further 4 weeks using the assigned toothbrush under the same routine as in phase 1.

To measure compliance, all subjects were asked to keep a self-produced checklist to record whether or not they brushed. Since they were instructed to brush at least twice a day, compliance was calculated as follows: compliance = (total number of toothbrushing sessions) × 100 / (28 days × 2 times per day).

### Randomization and blinding

Randomization was conducted using a computer-based random number allocation table (sealedenvelope. com). A random sequence was generated in blocks of 12 (test = 6, control = 6). Based on the randomization scheme, an independent research assistant sealed 60 consecutive individual opaque envelopes containing a referral to either the intervention or the control group. Each participant who signed the informed consent form was assigned a sequential number. The research assistants opened the envelopes in sequence, according to the subjects’ random numbers. The trial manager regularly checked for adherence to the instructions. The envelopes were stored in a locked cabinet that was not accessible to the study statistician or the investigators. The subject opened the envelope only after receiving the fitness test results and was not allowed to change groups after randomization. After randomization, neither the researcher nor statistician was blinded to the group assignments because of the nature of the intervention. However, the experimental groups were blinded to the participants.

### Study devices

**Fig. 1:**
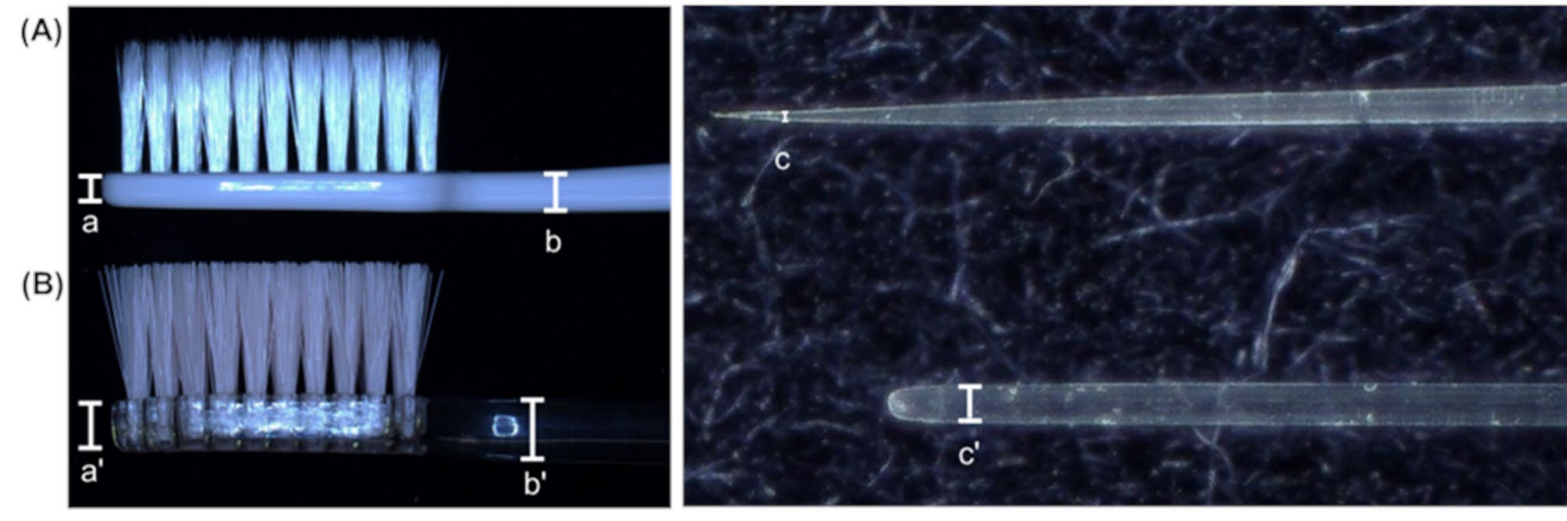
Brush head profile and bristle shape of study toothbrushes (**A**: test toothbrush, **B**: control toothbrush) (**a**) a’ head thickness; (**b**) b’ neck thickness; (**c**) c’ tip diameter of the filament

The toothbrush used in the test group was made of polybutylene terephthalate (PBT) as the primary resin and a thermoplastic elastomer (TPE) as the secondary resin, with 960 bristles. The test toothbrush had a head thickness of 3.0 mm, neck thickness of 3.5 mm and a bristle tip diameter of 0.02 mm (Fig. 1A). The toothbrush in the control group was composed of polycyclohexylene dimethylene terephthalate (PCTG) as the primary resin with 864 bristles. The control toothbrush had a head thickness of 4.5 mm, a neck thickness of 5.5 mm and a bristle tip diameter of 0.18 mm (Fig. 1B). Both toothbrushes had bristles that were 7 mm long and had a base diameter of 0.18 mm.

### Evaluation

#### Data collection

At baseline, all subjects were examined for dental caries (presence of caries and restorations), dental plaque and gingivitis. Photographs were taken using the QLF devices to quantitatively assess the deposition of the dental plaque around the teeth. At the end of phase-1, dental plaque and gingivitis checks were performed, and intraoral photographs were taken using the QLF devices. The same evaluations were performed at the beginning and end of phase-2. At the last visit, patients were asked to complete a self-administered questionnaire that included questions for evaluating the convenience of and satisfaction with using the assigned toothbrush. All oral examinations were performed by a periodontist at Yonsei University College of Dentistry.

#### Dental plaque evaluation

Dental plaque was assessed using two methods: the Silness-Loe plaque index (PI) and the plaque fluorescence test. PI was measured for teeth without restorations, being scored at six sites (distobuccal, midbuccal, mesiobuccal, distolingual, midlingual and mesiolingual) of each tooth following the criteria established by Silness and Lӧe [22]. The mean of the PI scores of the examined teeth was used as a representative value for the individual.

The plaque fluorescence tests were used to evaluate the activity of dental plaque using biofluorescence. These examinations were performed on anterior teeth and rearmost molars using two different devices. Fluorescence images of the anterior teeth were taken using a digital camera (Qraycam pro, AIOBIO, Seoul, Republic of Korea). The percentage of the dental plaque area detected by red fluorescence on the upper and lower anterior teeth in the photographs was calculated to evaluate the anterior dental plaque deposition.

For the rearmost molars the plaque fluorescence test was performed using an intraoral camera (Qraypen C, AIOBIO, Seoul, Republic of Korea) to capture images of the distal surfaces. The captured images were assigned the Simple Plaque Score (ranging from 0 to 5) provided by the Qray software. Subjects with challenging oral structures or restorations, or with images of insufficient resolution were excluded from the fluorescence analysis. Among the total of 58 participants, 52 were included in the analysis of anterior teeth and 34 in that of the rearmost molars. PI and plaque fluorescence tests were performed at baseline and 4 weeks later.

#### Gingivitis evaluation

The Lӧe-Silness gingival index (GI) was evaluated for index teeth (#12, 16, 24, 32, 36 and 44), implants and the rearmost molars. GI was scored at six sites (distobuccal, midbuccal, mesiobuccal, distolingual, midlingual and mesiolingual) of each tooth according to the criteria reported by Lӧe and Silness [22]. The mean GI score of examined tooth surfaces was used as the overall value for an individual.

The bleeding-on-probing index (BOP) was also measured in the same areas. The presence or absence of bleeding at 10–30 s after probing was evaluated. BOP was scored as 0 and 1 for the absence and presence of bleeding, respectively. GI and BOP were measured at baseline and 4 weeks later.

#### Survey of satisfaction with toothbrush usage

A self-administered survey developed for this study was used to assess the convenience of and satisfaction with using each toothbrush (see Supplementary Material). The survey responses were based on a five-point scale ranging from 1 point for “strongly disagree” to 5 points for “strongly agree”, with a higher score indicating a more positive response.

### Statistical analysis

All evaluation variables (dental plaque evaluation, gingivitis evaluation and survey results) were compared between the control and test toothbrushes, but not between the randomized groups. All results were presented as mean and standard deviation. The mean values of the changes in each variable for each subject after using toothbrushes were provided. The normality of the data was first assessed using the Shapiro-Wilk test. Based on the results, either a paired t-test or Wilcoxon signed-rank test was applied for group comparisons, with the significance values determined accordingly. All statistical analyses were conducted using SAS^®^ (Version 9.4, SAS Institute, Cary, North Carolina, USA). The level of statistical significance was set at *P* < 0.05.

## Results

**Fig. 2:**
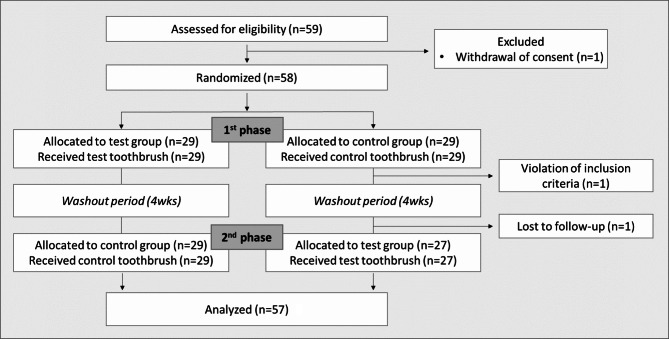
Flow chart according to the CONSORT guidelines. One control group participant was excluded for inclusion violation, and another participant lost to follow-up was included in the final analysis (Full Analysis Set)

Of the 59 participants assessed for eligibility, one withdrew consent, resulting in 58 participants being randomized into two groups for the first phase. Subsequently, one participant was excluded due to a violation of the inclusion criteria, and another was lost to follow-up, leaving 56 participants in the second phase. The remaining 56 participants were included in the final analysis (Fig. 2). All demographic characteristics, including sex and age, showed no significant differences between groups at baseline (*P* > 0.05; Table 1). No adverse events were reported during the follow-up period.

**Table 1:** Baseline demographic and background characteristics

		Group A (Test-Control) *n* = 29	Group B (Control-Test) *n* = 28	Total	*P*-value
Sex					
	Male	10 (34.48)	8 (28.57)	18 (31.58)	0.6312^†^
	Female	19 (65.52)	20 (71.43)	39 (68.42)	
Age		50.17 ± 15.60	56.36 ± 12.48	53.21 ± 14.37	0.1048^*^
Smoking					
	Never	23 (79.31)	27 (96.43)	50 (87.72)	0.1173^‡^
	Current	2 (6.90)	0 (0.00)	2 (3.51)	
	Former	4 (13.79)	1 (3.57)	5 (8.77)	
Alcohol consumption					
	Never	18 (62.07)	16 (57.14)	34 (59.65)	0.5602^‡^
	Former	2 (6.90)	2 (7.14)	4 (7.02)	
	< 1 bottle (/week)	8 (27.59)	6 (21.43)	14 (24.56)	
	> 1 bottle (/week)	1 (3.45)	4 (14.29)	5 (8.77)	
Monthly income (10,000 KRW)					
	< 300	8 (27.59)	7 (25.00)	15 (26.32)	0.9228^†^
	300–600	14 (48.28)	15 (53.57)	29 (50.88)	
	600<	7 (24.14)	6 (21.43)	13 (22.81)	
Education					
	< Middle school	3 (10.34)	3 (10.71)	6 (10.53)	0.9193^‡^
	High school	6 (20.69)	7 (25.00)	13 (22.81)	
	University≤	20 (68.97)	18 (64.29)	38 (66.67)	

^*^: *P*-value for Two sample t-test

^#^: *P*-value for Wilcoxon rank sum test

^†^: *P*-value for Chi-square test

^‡^: *P*-value for Fisher’s exact test

The compliance rates from the self-produced checklists for the test and control toothbrushes were 112.53 and 110.87, respectively, with no significant difference (*P =* 0.6421).

### Changes in clinical parameters of all teeth

After 4 weeks of test toothbrush use, PI, GI and BOP had decreased by 25%, 30% and 48%, respectively, across all teeth, with all of these differences being significant (all *P* < 0.0001, Table 2). In contrast, there were no significant reductions in PI, GI or BOP after using the control toothbrush (*P* > 0.05). The mean change in PI differed significantly between after using the test toothbrush and the control toothbrush (*P =* 0.0135). The mean changes in GI after using the test toothbrush was 0.31, while the mean change in BOP was 0.13, which were both significantly different from the mean changes after using the control toothbrush (*P =* 0.0146 and 0.0243, respectively).

**Table 2:** Comparison of PI, GI, and BOP in total teeth before and after using the two toothbrushes

		Test toothbrush (*n* = 57)				Control toothbrush (*n* = 57)		
		Baseline	4 weeks	*P*-value		Baseline	4 weeks	*P*-value
PI		0.53 ± 0.30	0.40 ± 0.32	< 0.0001^a)^		0.48 ± 0.30	0.48 ± 0.27	0.9624
	Mean differences	-0.12 ± 0.26				0.00 ± 0.25		0.0135
GI		1.04 ± 0.45	0.73 ± 0.39	< 0.0001		1.03 ± 0.38	0.95 ± 0.37	0.1552
	Mean differences	-0.31 ± 0.43				-0.08 ± 0.43		0.0146
BOP		0.27 ± 0.22	0.14 ± 0.15	< 0.0001		0.25 ± 0.19	0.23 ± 0.19	0.5498
	Mean differences	-0.13 ± 0.20				-0.02 ± 0.23		0.0243

PI: plaque index, GI: gingival index, BOP: bleeding on probing

*P*-values were calculated based on the Paired t-test except for ^a)^

^a)^: *P*-value for Wilcoxon signed rank test

### Changes in clinical parameters of the rearmost molars

PI decreased significantly by 18% after 4 weeks of using the test toothbrush for the rearmost molars (*P* = 0.0209, Table 3). In particular, GI and BOP—which are indicators of gingivitis—showed significant reductions of 26% and 47%, respectively, after using the test toothbrush (*P* < 0.0001). In contrast, there was no significant reduction in PI, GI or BOP after using the control toothbrush (*P* > 0.05). The mean change in PI did not differ significantly between after using the test toothbrush and the control toothbrush (*P* = 0.0756). Although the mean changes in GI and BOP after using the test toothbrush were approximately 5 times and 4 times larger, respectively than after using the control toothbrush, only the difference in GI was significant (*P* = 0.0349).

**Table 3:** Comparison of PI, GI, and BOP in rearmost teeth before and after using the two toothbrushes

		Test toothbrush (*n* = 57)				Control toothbrush (*n* = 57)		
		Baseline	4 weeks	*P*-value		Baseline	4 weeks	*P*-value
PI		0.67 ± 0.47	0.55 ± 0.42	0.0209		0.68 ± 0.37	0.68 ± 0.43	0.9607
	Mean differences	-0.12 ± 0.34				-0.00 ± 0.35		0.0756
GI		1.19 ± 0.51	0.88 ± 0.43	< 0.0001^a)^		1.21 ± 0.45	1.14 ± 0.45	0.3261
	*Mean differences*	-0.30 ± 0.51				-0.06 ± 0.48		0.0349
BOP		0.36 ± 0.28	0.19 ± 0.21	< 0.0001		0.38 ± 0.25	0.34 ± 0.28	0.3619
	*Mean differences*	-0.16 ± 0.27				-0.04 ± 0.32		0.0628

PI: plaque index, GI: gingival index, BOP: bleeding on probing

*P*-values were calculated based on the Paired t-test except for ^a)^

^a)^: *P*-value for Wilcoxon signed rank test

### Gingivitis changes in implants

For implants, after 4 weeks of using the test toothbrush there was a significant reduction of 31% in GI, from 1.05 to 0.72 (*P* < 0.0001, Table 4). In contrast, after using the control toothbrush GI showed a slight increasing trend from 1.04 to 1.10, which was not significant (*P* = 0.9033). BOP after using the test toothbrush decreased by approximately 57%, from 0.28 to 0.12 (*P* = 0.0002). In contrast, after using the control toothbrush BOP showed a tendency to increase from 0.25 to 0.33, but this also was not significant (*P* = 0.1722). The mean changes in GI and BOP after using the test toothbrush were both significantly different from those after using the control toothbrush (*P* = 0.0029 and 0.0011, respectively).

**Table 4:** Comparison of GI and BOP in implants before and after using the two toothbrushes

		Test toothbrush (*n* = 57)				Control toothbrush (*n* = 57)		
		Baseline	4 weeks	*P*-value		Baseline	4 weeks	*P*-value
GI		1.05 ± 0.54	0.72 ± 0.52	< 0.0001		1.04 ± 0.51	1.05 ± 0.64	0.9033
	*Mean differences*	-0.33 ± 0.57				0.01 ± 0.61		0.0029
BOP		0.28 ± 0.31	0.12 ± 0.21	0.0002^a)^		0.25 ± 0.32	0.33 ± 0.36	0.1722^a)^
	*Mean differences*	-0.15 ± 0.31				0.08 ± 0.37		0.0011^a)^

GI: gingival index, BOP: bleeding on probing

*P*-values were calculated based on the Paired t-test except for ^a)^

^a)^: *P*-value for Wilcoxon signed rank test

### Changes in plaque fluorescence of anterior teeth and rearmost molars

**Fig. 3:**
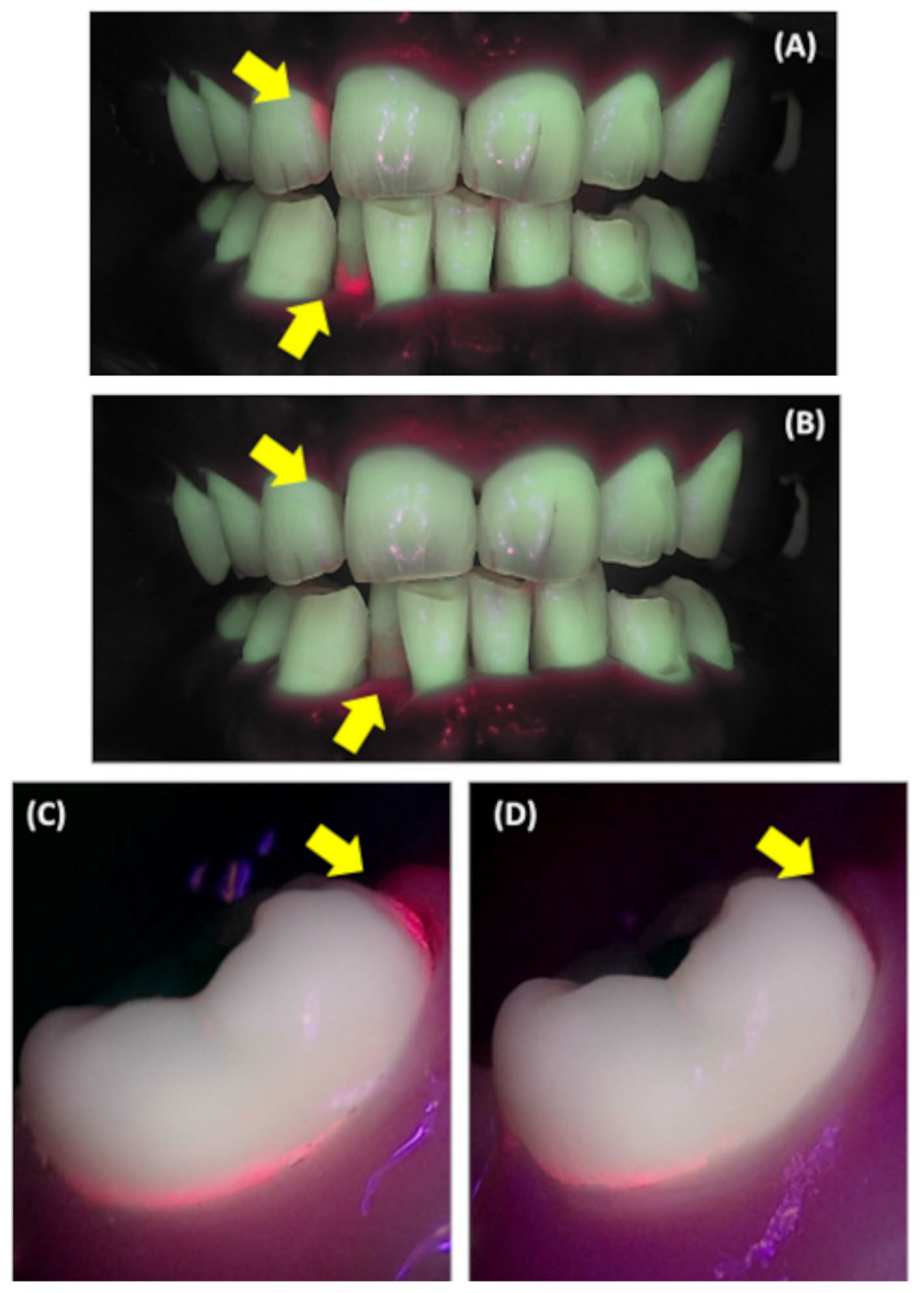
Representative Quantitative light-induced fluorescence images. (**A**) and (**C**) are the baseline; (**B**) and (**D**) are the end of the study

The evaluations of plaque fluorescence of the anterior teeth (Fig. 3A, B) after 4 weeks of using the test toothbrush showed that the area of dental plaque deposition decreased significantly by approximately 25%, from 0.51 to 0.38 (*P* = 0.0066, Table 5). In contrast, after using the control toothbrush the dental plaque area for the anterior teeth tended to decrease from 0.48 to 0.38, which was not significant (*P* = 0.1081). The mean change after using the test toothbrush was 0.13, which was larger than the mean change after using the control toothbrush of 0.09 (*P* = 0.9891). Although the average change showed a substantial trend, no significant differences were observed. The fluorescence evaluation of dental plaque on the rearmost molars (Fig. 3C, D) after using the test toothbrush revealed a tendency for a 14% reduction in dental plaque deposition on the distal surfaces of the rearmost molars (*P* = 0.5272); however, no significant difference was observed. The mean change after using the test toothbrush tended to be about 4 times higher than that after using the control toothbrush, but this difference was not significant (*P* = 0.1225).

**Table 5:** Comparison of plaque fluorescence in anterior and rearmost teeth before and after using the two toothbrushes

		Test toothbrush				Control toothbrush		
		Baseline	4 weeks	*P*-value		Baseline	4 weeks	*P*-value
Anterior teeth (*n* = 85)		0.51 ± 0.51	0.38 ± 0.39	0.0066		0.48 ± 0.54	0.38 ± 0.39	0.1081
	*Mean differences*	-0.13 ± 0.32				-0.09 ± 0.40		0.9891
Rearmost molars (*n* = 69)		1.59 ± 1.86	1.37 ± 1.58	0.5272		1.70 ± 1.82	1.76 ± 1.90	0.9318
	*Mean differences*	-0.22 ± 1.46				0.06 ± 1.60		0.1225

*P*-value for Wilcoxon signed rank test

Anterior teeth: Test toothbrush (*n* = 52), Control toothbrush (*n* = 53)

Rearmost molars: Test toothbrush (*n* = 34), Control toothbrush (*n* = 35)

### Satisfaction with and convenience of using the toothbrushes

The test toothbrush scored significantly higher than the control toothbrush on all the questions in the satisfaction and convenience survey (*P* < 0.05, Table 6). Specifically, the test toothbrush showed higher scores than the control toothbrush for satisfaction with using the head softness, neck flexibility and overall ease of use (*P* < 0.0001). Additionally, the test toothbrush scored approximately 1.2 times higher than the control toothbrush for the satisfaction questions of “feeling of cleaning thoroughly in all corners” and “feeling of effectively cleaning the rearmost molars” (*P* < 0.0001).

**Table 6:** Satisfaction and convenience of toothbrushes

	Test toothbrush	Control toothbrush	*P*-value
Level of satisfaction	3.88 ± 0.90	3.28 ± 1.11	0.0026
Level of convenience	4.13 ± 0.76	3.23 ± 1.09	< 0.0001
Feeling of cleaning thoroughly in all corners	4.07 ± 0.93	3.35 ± 1.11	< 0.0001
Feeling of effectively cleaning the rearmost molars	3.91 ± 0.88	3.19 ± 1.11	< 0.0001
Softness of bristle	4.39 ± 0.53	2.96 ± 1.27	< 0.0001
Bristle elasticity	3.98 ± 0.82	3.37 ± 0.96	0.0002
Toothbrush head size	4.05 ± 0.82	3.30 ± 1.00	< 0.0001
Toothbrush neck thickness	4.05 ± 0.75	3.44 ± 0.93	< 0.0001
Toothbrush neck flexibility	4.05 ± 0.77	3.14 ± 1.03	< 0.0001
Willingness to continue using	3.82 ± 1.03	3.09 ± 1.12	0.0002

*P*-value for Wilcoxon signed rank test

## Discussion

This study found that the toothbrush developed with a thin head, a slender neck and super-tapered bristles demonstrated superior effects on reducing dental plaque and gingivitis compared with the control toothbrush. The newly developed toothbrush was also found to be more convenient to use than the control toothbrush. In particular, excellent effects of the test toothbrush on dental plaque and gingivitis reduction were confirmed around the rearmost molars and the implant sites. Based on these findings, the null hypothesis was rejected, indicating a significant difference in the effects of the newly developed toothbrush compared with the control toothbrush.

The findings of evaluations of toothbrush efficacy may vary with the characteristics of the study population, since the efficacy of dental plaque removal depends on factors that vary between individuals, including the duration, method and intensity of brushing [15]. The crossover design used in the present study had the advantage of comparing conditions between before and after toothbrush use in the same patient, which results in less variation in an observed effect compared with a parallel design that compares results for different patients [23]. The present study also had the advantage of being conducted under real-use conditions with the subjects performing their usual brushing habits.

After 4 weeks of using the test toothbrush, significant decreases of approximately 25% in PI, 30% in GI and 48% in BOP were observed across the teeth of all of the participants (*P* < 0.05). Several previous studies have found tapered bristles to be more effective at removing dental plaque than traditional end-rounded toothbrushes [13, 14, 24, 25]. In a single-blind clinical study of the effectiveness of tapered-bristle toothbrushes in reducing dental plaque and gingivitis, the PI reduction rate for tapered-bristle toothbrushes after 3 months was 24% [26], which is similar to the PI reduction rate in this study. In addition, an in vitro study evaluating accessibility and cleaning effectiveness found that toothbrushes with super-tapered bristles, which were also used in this study, were approximately 1.5 times better in terms of interproximal access and 4.4 times better at cleaning the gingival margin compared with toothbrushes with end-rounded bristles [14]. Together these findings suggest that the super-tapered toothbrushes used in this study achieved similar results to the 3-month results of the previous studies, despite the short usage period of 1 month, due to their superior accessibility and gingival margin cleaning.

A systematic review and meta-analysis found a significant difference in GI after using tapered-bristle toothbrushes, with a mean difference of 0.12 relative to using toothbrushes with end-rounded bristles [27]. Additionally, a randomized controlled clinical trial comparing ADA standard toothbrushes and tapered-bristle toothbrushes found a reduction rate of 11–12% in GI for tapered and cross-angled soft-bristle toothbrushes, compared with no significant change for ADA standard toothbrushes after 1 month of toothbrushing [13]. Tapered-bristle toothbrushes have been reported to provide greater subgingival access [28], and subgingival plaque is associated with gingivitis and periodontitis. An in vitro test evaluating the subgingival access of tapered-bristle toothbrushes found that tapered-bristle toothbrushes were effective in removing subgingival artificial plaque [24], and Yankell et al. [29] reported that double-tapered filament toothbrushes were more effective than ADA standard toothbrushes in removing both supragingival and subgingival artificial plaque. It can therefore be assumed that the tapered bristles that are more effective at removing subgingival plaque were also more effective in reducing gingivitis in the present study.

We observed significant reductions in PI, GI and BOP after using the test toothbrush even for the hard-to-reach areas of the rearmost molars. This is probably attributable to the improved accessibility provided by the toothbrush design. The test toothbrush used in this study had a head that was 33% thinner than that of the control toothbrush (thickness of 3.0 vs. 4.5 mm). Consequently, it appears that the test toothbrush facilitated access to narrow interdental spaces within the oral cavity, making it easier to brush the rearmost molars located deep within the oral cavity. In fact, the survey results for the participants indicated high satisfaction levels with using the test toothbrush for both the “feeling of cleaning thoroughly in all corners” and “feeling of effectively cleaning the rearmost molars” questions (*P* < 0.0001). Additionally, the satisfaction score for the “flexibility of the toothbrush” was 4.05 for the test toothbrush and 3.14 for the control toothbrush, which was a significant difference of more than 1 point. This is because the neck of the test toothbrush in this study was 36% thinner than the neck of the control toothbrush (thickness of 3.5 vs. 5.5 mm), and so the subjects experienced the neck of the toothbrush as being more flexible. A robot model study of brushing using standardized brushing forces showed that toothbrushes with flexible necks are more effective at removing dental plaque for three different brushing motions [16]. Additionally, when examined by tooth type, it was reported that cleaning was most effective for incisors, followed by wisdom teeth, canines, and premolars. Thus, increased neck flexibility may facilitate access to the posterior hard-to-reach teeth.

This study found significant reductions of 31% and 57% in the peri-implant GI and BOP, respectively, after using the test toothbrush. Implants are known to have deeper probing depths than healthy natural teeth [30]. The preclinical study of Montevecchi et al. [5] examined the ability of tapered bristles to penetrate the peri-implant sulcus in a plaster model and found that penetration ability was 8 times higher for tapered bristles than for cylindrical bristles. Therefore, the tapered bristles of the test toothbrush used in the present study appear to have increased the ability to remove dental plaque from the deep peri-implant sulcus, thereby alleviating gingivitis. This study further found that the peri-implant BOP was significantly reduced by approximately 57% from baseline after using the test toothbrush. However, the peri-implant BOP should be interpreted with caution. Although BOP is considered an essential tool in diagnosing periodontal disease, the peri-implant BOP may be exaggerated since it can be influenced by the induction of trauma due to differences in the anatomical characteristics of the implant and the natural tooth. In particular, traumatic bleeding caused by weak prosthetic contours and peri-implant mucosa may have complicated the ability to interpret the results of the present study [30]. Although the implant BOP is not a complete predictor of peri-implant disease, it is a key variable in determining gingival health, since the absence of bleeding on probing indicates a healthy condition [30]. The present study found that BOP and GI decreased significantly at the same time, suggesting that the gingiva became healthier. The peri-implant soft tissues show a stronger inflammatory response to dental plaque accumulation compared with the gingival soft tissues [31], and therefore require more-thorough dental plaque control. Despite the different anatomical characteristics of implants and natural teeth, most of the current instruments for implant hygiene are general oral care products [5]. Therefore, the use of specialized toothbrushes that provide improved access to the peri-implant area may be a good option for preventing peri-implant disease.

This study performed the QLF plaque fluorescence test—which can intuitively identify dental plaque—in addition to visual PI for dental plaque detection. The use of dental stains has the disadvantage of staining all protein-derived structures attached to the tooth surface, including the acquired pellicle, resulting in overestimation of the amount of dental plaque [32, 33]. Given the condition that all tooth surfaces and oral soft tissues are stained by the tooth coloring agent, it would be unpleasant for the patient to wash their teeth after an evaluation of stained dental plaque. However, the plaque fluorescence test using biofluorescence technology can detect dental plaque through red biofluorescence caused by a metabolite (porphyrin) secreted by bacteria without the use of a separate tooth stain. In addition to screening for the presence or absence of dental plaque, the intensity of red fluorescence can be used to assess dental plaque characteristics such as maturity and pathogenicity [34, 35]. In this study, the red fluorescence area of the anterior teeth decreased by approximately 25% after 4 weeks of using the test toothbrush (*P* = 0.0066), which was consistent with the PI results. The simple plaque score of the distal surfaces of the rearmost molars tended to decrease by approximately 14%; however, this was not significant (*P* = 0.384). The rearmost molars are where the most calculus forms due to persistent dental plaque deposition and poor toothbrush access. Dental plaque forms due to the calcification of unremoved biofilm and is known to contain large amounts of porphyrins, a metabolite of anaerobic bacteria, which exhibit strong red fluorescence during QLF plaque fluorescence test [36]. This means that QLF detects not only dental plaque but also calculus. It is therefore possible that calculus that could not be removed by toothbrushing was included in the fluorescence plaque score, which may explain why no significant reduction was observed.

The satisfaction and convenience evaluations of both the test and control toothbrushes performed in this study revealed that the test toothbrush produced superior clinical outcomes for all of the associated questions. The interest in and positive attitudes toward oral care products among patients can have a long-term positive impact on the goal of maintaining a healthy oral cavity [37]. Therefore, a toothbrush that patients are subjectively satisfied with can have a positive impact on its continued use, and so long-term effectiveness of the test toothbrush investigated in this study is anticipated.

However, since the test and control toothbrushes used in this study had different shapes, it is possible that the Hawthorne effect occurred despite the random allocation of toothbrushes to the participants. This is a typical limitation that arises in most clinical studies involving new types of toothbrushes. Additionally, the test toothbrush evaluated in this study possessed three specific structural features: a thin head, a slender neck and super-tapered bristles. Which of these specific features made the greatest contributions to reducing dental plaque and gingivitis should be investigated in future studies with a design that allows for the evaluation of each factor separately. Also, most of the participants in this study had mild dental plaque accumulation and gingivitis, and so it might be useful to evaluate the effectiveness of the test toothbrush in a population with more-severe oral disease in the future.

## Conclusion

Using the newly developed toothbrush resulted in significant reductions in dental plaque and gingivitis compared with the control toothbrush in this study, especially for the rearmost molars and the implants, where dental plaque control was inadequate with the conventional toothbrush. Consequently, toothbrushes with a thin head, a slender neck and super-tapered bristles may be effective in removing dental plaque and preventing gingivitis in implant patients as well as in individuals with general oral conditions.

## Electronic supplementary material

Below is the link to the electronic supplementary material.

Supplementary Material 1

## Data Availability

The data included in the present study are available from the corresponding author on reasonable request.

## Abbreviations

PI: Plaque Index
GI: Gingival Index
BOP: bleeding on probing
QLF: Quantitative light-induced fluorescence
