# Supplementary material for: A novel toothbrush with a thin-head, slender-neck and super-tapered bristles enhancing accessibility in hard-to-reach areas: a crossover randomized trial
Source: BMC Oral Health. 2024 Oct 5;24:1186. doi: 10.1186/s12903-024-04975-3 (PMC11452929; doi:10.1186/s12903-024-04975-3)
Supplement: Supplementary file 1 — Supplementary Material 1 [file 12903_2024_4975_MOESM1_ESM.docx]

1. Are you satisfied with the toothbrush used during the study period?

□ Strongly disagree □ Disagree □ Neutral □ Agree □ Strongly agree

2. Is the use of this toothbrush more convenient?
□ Strongly disagree □ Disagree □ Neutral □ Agree □ Strongly agree

3. Does it feel like your entire mouth thoroughly clean?
□ Strongly disagree □ Disagree □ Neutral □ Agree □ Strongly agree

4. Does your rearmost molars feel well-cleaned?
□ Strongly disagree □ Disagree □ Neutral □ Agree □ Strongly agree

5. Did the bristles of the toothbrush feel soft when used?
□ Strongly disagree □ Disagree □ Neutral □ Agree □ Strongly agree

6. How would you rate the elasticity of the bristles of the toothbrush?

□ Very not good □ Not good □ Average □ Good □ Very good

7. How do you feel about the size of the toothbrush head?
□ Very not good □ Not good □ Average □ Good □ Very good

8. How do you feel about the thickness of the toothbrush neck?
□ Very not good □ Not good □ Average □ Good □ Very good

9. How would you rate the flexibility of the toothbrush neck?
□ Very not good □ Not good □ Average □ Good □ Very good

10. Do you plan to continue using this product in the future?

□ Strongly disagree □ Disagree □ Neutral □ Agree □ Strongly agree
